# *N*-Acyl Homoserine Lactone-Mediated Quorum Sensing with Special Reference to Use of Quorum Quenching Bacteria in Membrane Biofouling Control

**DOI:** 10.1155/2014/162584

**Published:** 2014-07-24

**Authors:** Harshad Lade, Diby Paul, Ji Hyang Kweon

**Affiliations:** Department of Environmental Engineering, Konkuk University, Seoul 143-701, Republic of Korea

## Abstract

Membrane biofouling remains a severe problem to be addressed in wastewater treatment systems affecting reactor performance and economy. The finding that many wastewater bacteria rely on *N*-acyl homoserine lactone-mediated quorum sensing to synchronize their activities essential for biofilm formations; the quenching bacterial quorum sensing suggests a promising approach for control of membrane biofouling. A variety of quorum quenching compounds of both synthetic and natural origin have been identified and found effective in inhibition of membrane biofouling with much less environmental impact than traditional antimicrobials. Work over the past few years has demonstrated that enzymatic quorum quenching mechanisms are widely conserved in several prokaryotic organisms and can be utilized as a potent tool for inhibition of membrane biofouling. Such naturally occurring bacterial quorum quenching mechanisms also play important roles in microbe-microbe interactions and have been used to develop sustainable nonantibiotic antifouling strategies. Advances in membrane fabrication and bacteria entrapment techniques have allowed the implication of such quorum quenching bacteria for better design of membrane bioreactor with improved antibiofouling efficacies. In view of this, the present paper is designed to review and discuss the recent developments in control of membrane biofouling with special emphasis on quorum quenching bacteria that are applied in membrane bioreactors.

## 1. Introduction

Advanced wastewater treatment technology membrane bioreactor (MBR) combines the use of biological degradation process by activated sludge with a direct solid-liquid separation by micro- or ultrafiltration membranes of pore sizes ranging from 0.05 to 0.4 *μ*m [1]. These membranes allow the complete retention of bacteria and suspended solids within bioreactor and only water with reusable quality gets released. As a result, the MBRs are increasingly emerging as an advanced treatment processes in industrial and municipal wastewaters [2]. Even though MBRs have been implemented in commercial applications for more than two decades, one of the major problems restricting their wide spread use is membrane biofouling [3]. Membrane biofouling is the accumulation of microorganisms and their metabolites produced such as extracellular polymeric substances (EPS) on membrane surfaces. This results in the unacceptable operational problems like decay in filtrate flux, pressure drop increase, and, thus, alteration on membranes [4]. The biofouling problem progresses throughout the treatment process, so the membranes have to be cleaned and eventually replaced [5]. Thus, biofouling is considered as the most severe problem in wastewater treatment systems resulting in greater loss in the economy.

Different physicochemical and biological practices have been tried to overcome the problem of membrane biofouling. These include physical cleaning of membrane surfaces with hot water, fabrication of biofouling resistant membranes, and incorporation of antibiotics or antimicrobial compounds in MBR [1, 6]. Indiscriminate use of antibiotics puts selection pressure on bacteria by interfering with their vital genomic functions like protein synthesis, RNA synthesis, and DNA synthesis [7, 8]. These result in the emergence of multidrug resistance among pathogenic bacteria and thus are considered as a serious and growing phenomenon in contemporary medicines used in human healthcare. In this scenario, it is necessary to use alternative approach to replace antibiotics for combating membrane biofouling.

Studies to mitigate membrane biofouling have suggested that biofilm formation is mostly associated with Gram-negative bacteria and their secreted metabolites [9]. Several species of Gram-negative bacteria communicate by synthesizing, secreting, and responding to small diffusible signal molecules* N*-acyl homoserine lactones (AHLs) through a mechanism called quorum sensing [10, 11]. The AHLs-mediated cell-to-cell signaling allows these bacteria to coordinate gene expression and regulate different phenotypes such as biofilm formation, secretion of EPS, and virulence factor [12–14]. Moreover, the AHL-mediated quorum sensing system is associated with almost all stages of biofilm formation such as initial surface attachment, bacterial growth, maturation, and detachment of aged cells [15, 16].

As quorum sensing plays significant roles in the establishment of biofilms by Gram-negative bacteria, disruption of AHL-based signaling has become the promising strategy to control membrane biofouling [17]. Three targets that can intercept AHL-based quorum sensing and modulate its controlled behaviors like biofilm formation are known, which include (i) inhibition of AHL synthesis by blocking synthase proteins [18], (ii) interference with signal receptors [19], and (iii) enzymatic degradation or alteration of AHLs molecules [20, 21]. Recently, some natural compounds such as vanillin, furanones, and curcumin have been found to intercept AHL-mediated quorum sensing system and thus inhibit membrane biofouling [22–24]. However, this approach is not feasible to use at commercial levels due to higher cost of natural compounds and the fact that more doses are required to achieve considerable biofouling inhibition. Another promising approach is that the enzymatic quorum quenching (in the form of a free enzyme or an immobilized form on a bead) has been successfully applied to mitigate biofouling in submerged membrane bioreactor treating wastewaters [25, 26]. But the higher cost of purified enzymes makes it difficult to use at commercial levels.

In view of this, a novel biological paradigm with the application of quorum quenching bacteria in MBRs has been investigated recently. This has proven more effective and economically feasible with membrane encapsulated and bead entrapped quorum quenching bacterial studies and suggested a new milestone towards widespread antibiofouling applications. Thus, in this review, we briefly overview the AHL-mediated biofilm formations by Gram-negative bacteria and elucidate the roles of enzymatic interference of AHLs by quorum quenching bacteria in inhibiting membrane biofouling.

## 2. Quorum Sensing for Coordinated Behaviors in Bacteria

Many Gram-positive and Gram-negative bacteria use quorum sensing signal circuits to coordinate a diverse array of physiological behaviors such as symbiosis, competence, virulence, conjugation, antibiotic production, sporulation, motility, and biofilm formation [27]. The quorum sensing system has been divided into two paradigmatic classes: oligopeptide/two component-type quorum sensing circuits in Gram-positive bacteria and* Lux I*/*Lux R*-type quorum sensing system in Gram-negative bacteria [28]. The difference in regulatory process depends on the chemical structure of signal molecule and its detection mechanism [29]. In general, Gram-positive bacteria use processed oligopeptides and Gram-negative bacteria use AHL as signal molecule to coordinate their behaviors. Furthermore, the molecular bases of the synthesis and perception of different quorum sensing signals and details of the signal transduction pathways have revealed their specific behaviors. As AHL-mediated quorum sensing system of Gram-negative bacteria is known to be involved in biofilm formations, we will only briefly address its mechanisms.

### 2.1. AHL-Mediated Quorum Sensing

Predominant Gram-negative Proteobacteria belonging to *α*, *β*, and *γ* subdivisions utilize AHL-mediated quorum sensing pathways to regulate their behaviors [30]. However, Gram-positive bacteria belonging to the* Exiguobacterium* genera have been recently identified as AHL producer [31]. AHLs are amphipathic in nature and are soluble in water and freely diffusible through cell membranes [32, 33].

**Figure 1:**
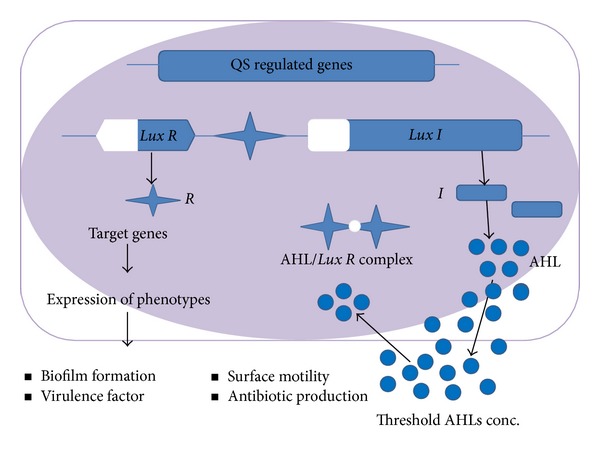
Schematic representation of the LuxR/AHL type quorum sensing system in Gram-negative bacteria. The “r” is a gene encoding* Lux R*-type transcription factor R and “i” is gene encoding* Lux I*-type AHL synthase I. Transcription of QS-regulated target genes appears by* Lux R* homologue proteins only when high AHL concentration is present, which required a threshold bacterial cell density.

The AHL-mediated quorum sensing system requires three major components to function: (i) the AHL signal molecule, (ii) AHL synthase protein to make the AHL signal, and (iii) a regulatory protein which responds to the surrounding concentration of AHLs [34]. A schematic representation of the AHL-mediated quorum sensing is shown in Figure 1. In AHL-mediated quorum sensing, a single synthase-regulator complex is responsible for the expression of specific genes. The signal molecules are produced by an AHL synthase gene* Lux I* at low concentration and are distributed in and around the cell. At lower cell densities, the* Lux I* is constitutively expressed at a low, basal level and thus AHLs get accumulated in the surrounding [35]. At high concentration of AHLs, the signal-receptor protein complex forms and gets activated. The activated signal-receptor complex in turn forms dimers or multimers with other activated AHL-*lux R* complexes and functions as transcriptional regulators controlling the expression of quorum sensing regulated target genes. At a certain cell density, also known as “quorum size,” the transcription of quorum sensing genes gets triggered and results in the expression of various phenotypes [36, 37]. Each individual quorum sensing regulated gene has its own specific quorum size to activate and there is no single population density at which all the genes are activated [38, 39].

AHL-mediated quorum sensing is the most widely studied and best understood model of cell-to-cell communication in Gram-negative bacteria regulating various phenotypes. In* Pseudomonas aeruginosa* PAO1, swarming motility, expression of virulence factors, and biofilm maturation are regulated by AHL-mediated quorum sensing system [40]. The AHL-based quorum sensing in* Serratia liquefaciens *regulates swarming motility which results in the maturation of heterogeneous biofilms [41].* Burkholderia cepacia*, a common bacteria found in the membrane systems, has been shown to use cepI/R quorum sensing to control biofilm maturation [42]. In a natural inhabitant of waters,* Vibrio cholerae*, the transcriptional regulator hapRAHL, has been shown to be responsible for EPS synthesis and biofilm formation [43]. Moreover, some other Gram-negative bacteria, v.z.* Aeromonas hydrophila, P. aeruginosa*, and so forth, have been shown to use AHL-based quorum sensing system to regulate numerous phenotypes including biofilm formation [22, 44].

### 2.2. AHLs Production and Phenotypes Controlled

The AHLs which have been characterized so far consist of homoserine lactone (HSL) ring unsubstituted in the *β*- and*γ*-positions which is* N*-acylated with a fatty acyl group at the*α*-position [154]. The naturally occurring AHLs produced by Gram-negative bacteria exhibit varying lengths of acyl chain with 4 to 18 carbon atoms and contain either* N*-acyl,* N*-(3-oxoacyl), or* N*-(3-hydroxyacyl) classes [10, 73]. Some AHLs with unsaturation in the 5 and 7 positions in a chain of 12 or 14 carbon atoms have been also reported. The screening for putative AHL producers has revealed that Gram-negative bacteria belonging to different genera which occupy a wide variety of environmental sources produce AHLs. Some examples of AHLs producing bacteria include species of* Acinetobacter*,* Aeromonas*,* Agrobacterium*,* Burkholderia*,* Erwinia*,* Enterobacter*,* Chromobacterium*,* Methylobacter*,* Paracoccus*,* Pseudomonas*,* Ralstonia*,* Rhodobacter*,* Rhizobium*,* Serratia*,* Sinorhizobium*,* Vibrio*, and* Yersinia* [155]. Multiple AHLs have also been reported in these bacteria due to the presence of more than one AHL synthase. In addition, AHL signal production is a consequence of sloppy active site selection for the acyl chain and hence a single synthase will often make multiple AHL types. Thus, the presence of single or multiple AHL synthase in a single bacterium has resulted in the regulation of various quorum sensing phenotypes in one organism which includes virulence factor, exopolysaccharide production, swarming motility, antibiotic production, pigmentation, and biofilm formation. The detailed structural information of AHLs identified in Gram-negative bacteria and various phenotypes controlled is given in Table 1.

### 2.3. AHLs-Mediated Biofilm Formations in Wastewaters

Bacterial biofilms are present in many water and wastewater treatment systems, where they may play beneficial or detrimental roles [34]. Bacteria prefer to live in biofilms, as the mode of bacterial life in the form of biofilms confers many advantages over a planktonic mode of life, such as resistance to environmental stresses [156]. The formation of biofilms is a stepwise process involving the initial attachment of bacteria to surfaces, microcolonies growth and maturation into expanding structures, and further detachment of aged microorganisms [16]. In general, the transition from free-living individual cell to a sessile form initiated with the transportation and attachment of bacteria to specific substratum followed by adhesion, colonization, and setup of early biofilm structures [157]. It has been proposed that AHL-mediated quorum sensing system of Gram-negative bacteria is involved in almost all stages of biofilm formation such as swarming motility and dispersal of aggregates in* S. liquefaciens* and biofilm maturation in* P. aeruginosa* [15, 158, 159]. The influence of quorum sensing signal molecule 3-oxo-C12-HSL synthesis on biofilm maturation in* P. aeruginosa* has been described by Davies et al. [9]. It is also reported that quorum sensing regulated cell surface properties alteration seems to translate to a biofilm phenotype variation [15, 160]. The specific role of individual HSL in biofilm formation has been also reported, where C4-HSL was found to be involved in the initial surface attachment and maturation of* A. hydrophila* biofilms [161].

Several biofilm forming bacterial species have been identified in wastewater treatment systems and are known to possess AHL-mediated quorum sensing mechanism [162]. Moreover, a number of AHLs producing bacterial strains have been isolated from wastewaters and found to be involved in quorum sensing-mediated biofilm formation [88, 163–165]. Different AHLs have also been detected from wastewaters as produced by Gram-negative Proteobacteria belonging to *α*, *β*, and *γ* subdivisions [158]. Furthermore, a correlation between AHL production and biofilm formation has been found among wastewater bacterial isolates as accessed by biofilm formation assay [88]. It is suggested that, in Gram-negative bacterium* S. liquefaciens*, the AHL-mediated quorum sensing regulates swarming motility resulting in formations of heterogeneous biofilms [41]. The detection of C6-HSL and C8-HSL in the MBR biocake also indicates the involvement of AHLs producing bacteria in biofilm formation [17]. All these evidences suggest that the AHL-mediated quorum sensing system present in several Gram-negative wastewater bacteria is responsible for the formation of biofilms and thus by membrane biofouling.

The bacterial species identified in wastewater treatment systems possessing AHLs-mediated quorum sensing mechanisms are shown in Table 2. This list includes the only culturable bacteria that were identified in wastewater treatment systems and are involved in AHL-based membrane biofouling. However, the number of biofouling bacteria will obviously increase with further investigation of quorum sensing regulation and interspecies interaction. In addition to this, most of the wastewater bacteria are unculturable and have not been specifically studied so far to understand their genetic and physiological attributes. Advanced molecular biology techniques such as pyrosequencing will be used for detailed characterization of unculturable bacteria present in wastewater treatment systems and further study to understand the quorum sensing mechanism involved.

## 3. Quorum Quenching Disrupts Quorum Sensing Phenotypes

The mechanism that can interfere with any phenotype regulated by quorum sensing is known as quorum quenching [166]. There are three basic components and thus targets for external intervention in AHL-mediated quorum sensing system have been identified which include Lux I-type synthase which generates AHL signals, the AHL ligand as signal itself, and the Lux R-type signal receptor [18, 167, 168]. Among all these targets, the enzymatic degradation of AHL signal molecules has been reported in a wide range of prokaryotes and a few eukaryotes [169]. Thus, one of the most important prerequisites for designing quorum quenching strategies is the screening of Gram-negative bacteria for putative AHLs production. In view of this, the simple AHL biosensors bacterial strains based on* lux*,* lacZ,* or* gfp* reporter gene fusion or pigment induction have been developed which can be used to detect the presence of broad range of AHLs among Gram-negative bacteria [170].

### 3.1. Bacterial Biosensors for Detection of AHLs

**Figure 2:**
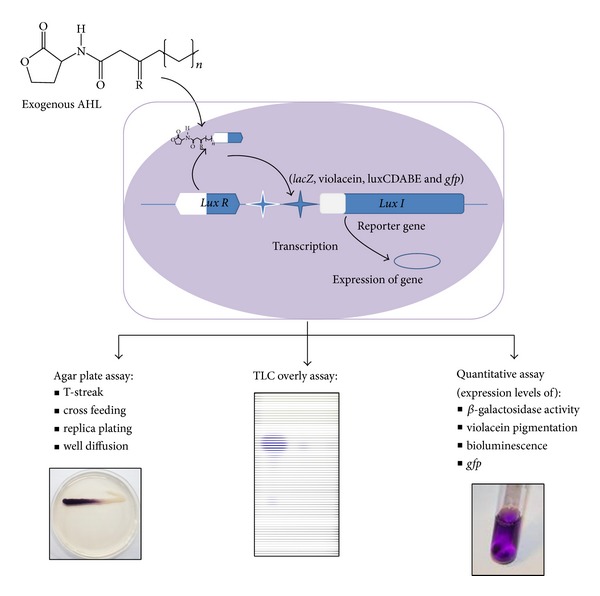
Construction of bacterial biosensor for the detection of exogenous AHLs. The bacterial biosensor is deficient in AHL production and when exogenous AHL interacts with* LuxR* protein, the transcription of reporter genes from* LuxR*-AHL regulated promoter initiated. This results in the display of specific phenotypes such as *β*-galactosidase activity, violacein pigmentation, bioluminescence, and green fluorescent protein production.

The detection of AHLs producing bacteria can be achieved by several methods. One common approach involving the use of biosensors strains is sensitive and convenient and allows real time detection of AHLs [171]. Biosensors strains contain quorum sensing regulatory promoters fused to* lux* operon or* lacZ* and lack AHL synthase enzyme. Such developed strains cannot produce AHLs but promoter activity gets induced by exogenous quorum sensing signals. Thus, the receptor gets activated and binds to its cognate* LuxI* promoter which initiates the expression of certain genes [45, 98]. The expression of relevant genes results in the display of specific phenotypes such as *β*-galactosidase production by* Ag. tumefaciens* NT1 [94], violacein pigmentation by* C. violaceum* CV026 [53], green fluorescent protein production by* V. fischeri *[105], and bioluminescence by* P. putida* 117 [101]. These biosensors strains can detect a narrow range of AHLs and thus more than one kind of such biosensors are required to test the wide range of AHLs produced by a single bacterium. Although biosensors were initially developed to detect the presence of AHLs in environmental isolates, they have also been used to investigate the activities of nonnative AHL analogues. The AHL detection bioassays are most commonly performed by overlay method while quantitative assays are performed by liquid cultures. A graphical representation for the construction of bacterial biosensor and its use to detect exogenous AHLs by means of different assay is shown in Figure 2.

The most commonly used biosensor strain* Ag. tumefaciens *NT1 (traR, tra::lacZ749) contains a lacZ fusion in the tra1 gene of pTiC58 which is induced to produce blue colour from the hydrolysis of 5-bromo-4-chloro-3-indolyl-*β*-D-galactopyranoside by the *β*-galactosidase activity, in response to broad range of AHLs such as 3-oxo-HSLs with side chains ranging from C4 to C12, 3-unsubstituted-HSLs with side chains from C6 to C12, and 3-hydroxy-HSLs with side chains from C8 to C10 [94, 172]. Another equally sensitive biosensor strain for long chain AHLs detection is* Ag. tumefaciens *A136. It contains the traI-lacZ fusion in the (pCF218) (pCF372) plasmids and is capable of detecting the presence C8-HSL, 3-oxo-C8-HSL, C10-HSL, C12-HSL, 3-oxo-C12-HSL, and C14-HSL exogenous AHLs by *β*-galactosidase activity [17, 37, 148]. The second class of reporter strain required for identifying short-chain AHLs with acyl chains of C4 to C6 is represented by* C. violaceum *CV026. It is mini-Tn5 mutant of ATCC31532 containing LuxR homologue CviR regulating the production of violacein, a purple pigment when induced by short-chain exogenous AHLs [53, 96]. A more recent developed reporter strain for detecting long-chain AHLs ranging from 3-oxo-C6-HSL to C14-HSL is* C. violaceum* VIR24, an in-frame deletion mutant of the* cvil* gene encoding AHL synthase in* C. violaceum* ATCC12472 [97]. The use of* P. putida* 117 as bioluminescence sensor for detection of medium-chain C8-HSL is suggested by Steidle et al. [101]. The green fluorescent protein derivative GFPmut3∗ and its unstable variant have also proven effective biosensors for detecting the presence of AHLs [105]. The GFPmut3∗ emits fluorescent light in the presence of oxygen and does not require any additional substrate. In addition to this, the plasmid sensor pSB1075 based on* Escherichia coli *bioluminescence has also been reported to detect the presence of C10-HSL, C12-HSL, and their 3-oxo derivatives [98]. In addition to the above-mentioned biosensors, new biosensors have also been developed so far and are summarized and listed in Table 3.

### 3.2. Quorum Quenching and Biofilm Control

A large number of molecules capable of disrupting AHL-mediated quorum sensing system have been identified and their mechanisms are revealed, which includes halogenated furanones produced by seaweed* Delisea pulchra* and synthetic derivatives that target R proteins [173], synthetic AHL analogues that may compete with corresponding AHL signals [174], and quorum quenching enzymes such as AHL-acylase, AHL-lactonase, and oxidoreductases which degrade or modify AHL signals [20, 128, 153]. Such quorum quenching compounds and enzymes with different mechanisms have been widely used in quenching AHL-mediated quorum sensing and thus preventing bacterial biofilms.

A detailed summary of the known natural quorum quenching molecules derived from plant, fungi, algae, and bacteria is provided in our previous review [175]. These compounds have been widely investigated in disease to combat AHL-mediated quorum sensing trait biofilm formations. However, very little research has been done so far using natural compounds on the inhibition of biofilm formations in advanced wastewater treatment systems. Recently, vanillin has shown to interfere with* A. hydrophila* quorum sensing and inhibited biofilm formations on five different membrane surfaces in a CDC (Center for Disease Control) biofilm reactor study [22]. Two more natural quorum sensing inhibitory compounds, furanones and* Piper betle*, have also been found to inhibit membrane biofouling in wastewater treatment systems [23, 176]. However, such purified natural compounds are not feasible to use at real MBRs due to the higher cost incurred for its extraction and purification, narrow efficacy towards specific AHLs, and high quantity required to achieve considerable biofouling inhibition. For example, vanillin showed the inhibition of only short-chain C4-HSL and C6-HSL and medium-chain 3-oxo-C8-HSL and C8-HSL AHLs, while it failed to inhibit long-chain AHLs [22]. Moreover, the quantity required to achieve considerable inhibition of AHLs is also high; that is, 0.25 mg/mL of vanillin showed the highest QSI activity with C4-HSL (69%) followed by 3-Oxo-C8-HSL (59.8%), C6-HSL (32%), and C8-HSL (28%). In addition, only 46.3% of biofilm inhibition was observed at the tested higher concentration of 0.25 mg/mL vanillin. The only major advantage of this novel strategy for antibiofouling method is that it circumvents the problem of resistance which is linked to the use antibiotics, as it specifically interferes with the expression of phenotypes rather than impede growth [137].

Another nonantibiotic approach studied to mitigate bacterial biofilms is the use of enzymes which can interfere with AHL signals and thereby inhibit its phenotypes. This approach of enzymatic quorum quenching has been attempted by many researchers to control membrane biofouling in MBRs treating wastewaters. Paul et al. [177] demonstrated the potential of purified AHL-degrading enzyme acylase I (porcine kidney) to reduce biofilm formations by environmental strains* A. hydrophila* and* P. putida* on three different membrane surfaces. To avoid the loss of free enzymes and maintain their stability, various methods of enzyme carriers have been tried. Recently, Yeon et al. [17] prepared a magnetic enzyme carrier by immobilizing quorum quenching enzyme acylase on magnetic particles to overcome the limitation of free enzyme and demonstrated its potential to control biofouling in MBR. In another study, the immobilization of acylase was carried out onto the membrane surface and mitigation of membrane biofouling investigated [26]. These innovative approaches of enzymatic quorum quenching have proven its potential for the control of biofouling in MBR treating wastewaters. However, some practical issues related to the high cost of purified enzymes and its instability make it difficult to use at commercial level MBRs treating municipal and industrial wastewaters. As an alternative to enzymatic quenching, the use of bacteria that produce quorum quenching enzymes and also help to decompose wastewater pollutant has been suggested [17, 148, 178].

## 4. Quorum Quenching Bacteria

The discovery of quorum quenching mechanisms in several bacterial species represents a new milestone in quorum sensing and quorum quenching research. Considering the essential roles of AHL-mediated quorum sensing in biofilm formation by Gram-negative bacteria, degradation or disruption of AHLs signals with quorum quenching enzymes produced by other bacteria appears to be a promising alterative for controlling membrane biofouling [179]. Therefore, strategies of disrupting the AHL-mediated quorum sensing with special emphasis on the control of membrane biofouling by quorum quenching bacteria are discussed herein.

**Figure 3:**
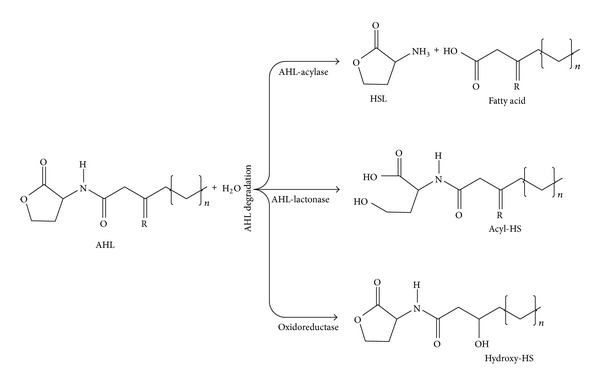
AHL-degradation or modification mechanism of quorum quenching enzymes: AHL-acylase, AHL-lactonase, and oxidoreductase.

Over the last few years, a range of quorum quenching enzymes have been identified in various Gram-negative and Gram-positive bacteria. These novel enzymes are key molecules for establishing the concept of quorum quenching in regulating quorum sensing phenotypes. The AHL-degrading or modifying enzymes are often classified into three groups: (i) AHL-acylases, (ii) AHL-lactonases, and (iii) oxidoreductases [20, 128, 153]. It has been known so far that four potential cleavage sites in the AHLs are likely cut off by quorum quenching enzymes following a catabolic digestion of carbon and nitrogen sources [124]. The crystal structural characterization of quorum quenching enzymes has also provided the valuable information to elucidate its catalytic mechanisms [166]. Additionally, the molecular biology techniques have identified the genes responsible for production of quorum quenching enzymes and its phenotypes regulated. The general mechanisms of these enzymes involved in the degradation or modification of AHL signals are shown in Figure 3.

AHL-acylases are known to irreversibly hydrolyze the amide linkage between the acyl chain and homoserine moiety of AHL signals resulting in the release of homoserine lactone and corresponding fatty acid, which do not exhibit further residual quorum sensing activity [20, 119]. The AHL-acylase was first reported in* V. paradoxus* strain VAI-C, which showed a wide range of degradation capacity against C4-HSL, 3-oxo-C6-HSL, C6-HSL, C8-HSL, C10-HSL, and C12-HSL [119]. Subsequently, several bacterial species have been reported to produce AHL-acylases such as AiiC in* Anabaena* sp. PCC7120 degrading C4-HSL to C14-HSL with 3-oxo and 3-hydroxy substitutions [106], QuiP in* P. aeruginosa* PAO1 degrading C8-HSL, C10-HSL, 3-oxo-C12-HSL, and C12-HSL [112], AiiD in* Ralstonia *sp. XJ12B degrading 3-oxo-C8-HSL, 3-oxo-C10-HSL, and 3-oxo-C12-HSL [20], and AhlM in* Streptomyces* sp. M664 degrading C8-HSL, C10-HSL, and 3-oxo-C12-HSL [117].

Another class of quorum quenching enzyme found in bacteria which degrades AHL molecule is AHL-lactonases [128]. This cleaves the homoserine lactone ring of AHLs in a hydrolytic and reversible manner to open the lactone ring, which makes the AHL incapable of binding to the target transcriptional regulator and attenuates its effectiveness [128]. The hydrolysis of lactone ring also appears at alkaline pH and can be reversed by acidification. Several AHL-lactonases have been identified from a range of bacterial species and are mentioned in some previous reviews [175, 180]. The first AHL-lactonase, encoded by aiiA gene of* Bacillus* sp. 240B1, was identified as AiiA^240B1^ by functional cloning of AHL signal as substrate in* E. coli* [128]. The AiiA^240B1^ has been shown to degrade C8-HSL and decreases the extracellular pectolytic enzyme activities and inhibition of virulence in* Er. carotovora.* It is reported that AiiA like lactonases hydrolytic activity is not affected by differences in the acyl chain length and substitutions in the AHLs [126, 181]. Another important class of AHL-lactonase is represented by the QsdA from* Rh. erythropolis* strain W2, which has been shown to degrade a wide range of AHLs including C6-HSL, C8-HSL, C10-HSL, C12-HSL, and C14-HSL with 3-oxo-substitutions [146]. The quorum quenching enzyme QsdA has been found to degrade the AHL-molecule and inhibits virulence factor in* Pec. carotovorum *strain PCC797. It is also reported that the QsdA lactonases belong to phosphotriesterase family which harbors lactonase, phosphotriesterase, or amidohydrolase activities [146]. Several other bacterial species including* Ochrobactrum *sp. T63,* Ag. tumefaciens *c58,* P. aeruginosa* PAO1, and* Bacillus *sp. 240B1 have been reported to encode AHL-acylase for degradation of AHLs which results in the inhibition of biofilm formation as listed in Table 4.

Oxidoreductase is the third important class of quorum quenching enzymes found in limited number of bacterial species. The oxidoreductases are known to target the acyl side chain by oxidative or reductive manner and thus catalyze the structural modification of AHL signal without degradation [182]. This structural change in AHL signal thus affects its specificity and recognition which results in the disturbance of the activation of quorum sensing-mediated phenotypes by modified AHL [114]. The bacterial oxidoreductases are suggested to oxidize a range of long-chain AHLs with or without 3-oxo-substitutions [115, 153]. The first bacterial oxidoreductase P450BM3 has been isolated from* Bacillus megaterium *which showed the oxidation of C12-HSL, 3-oxo-C12-HSL, C14-HSL, 3-oxo-C14-HSL, C16-HSL, C18-HSL, and C20-HSL [153]. Another unknown quorum quenching enzyme has been reported from* Rho. erythropolis* W2 which showed the oxidation of 3-oxo-C10 and 3-oxo-C12-HSL [115]. Additionally, one more enzyme was found in* Rho. erythropolis* W2 which can reduce the 3-oxo substituent of 3-oxo-C14-HSL to yield the corresponding derivative 3-hydroxy-C14-HSL and results in the inhibition of quorum sensing phenotypes.

All these enzymatic quorum quenching mechanisms present in bacteria could be used as a potent antibiofouling tool in MBRs treating wastewaters. A detailed survey of literature on quorum quenching bacteria has been carried out and some strains with AHLs degradation or modification activities are presented in Table 4.

### 4.1. Application of Quorum Quenching Bacteria in MBR

Enzymatic quorum quenching has proven its potential as an effective approach for biofouling control in the MBRs for advanced wastewater treatment [178]. Several groups of bacteria known to produce quorum quenching enzymes have also been reported and could be further elaborated as economically feasible antibiofouling tool in MBR. This interspecies quorum quenching mechanism present in bacterial cells will thus help to resolve the practical issues concerned with extraction and purification cost of free enzyme as well as its stability. In view of this, the practical applicability of quorum quenching bacteria in the regulation of biofilm formations in wastewater treatment systems has been investigated recently. This will provide valuable information in addressing both the basic and connectional problems associated with membrane biofouling.

Oh et al. [178] investigated the inhibition of quorum sensing in MBR by two quorum quenching bacteria, a recombinant* E. coli* which produces AHL-lactonase and a real MBR isolate* Rhodococcus *sp. A quorum quenching microbial vessel prepared by encapsulating both the bacterial strains into a microporous membrane (polyethylene hollow fiber) has successfully inhibited the membrane biofouling by interspecies interference in MBR treating wastewater. Moreover, the continuous MBR operation in the presence of inserted microbial vessel has also inhibited biofouling as determined by substantial delay in the TMP rise-up without any deterioration of wastewater treatment performance. In another study with* Rhodococcus *sp. BH4 encapsulated microbial vessel, the quorum quenching activity has been found to coincide well with biofouling inhibition in the continuous MBR [149]. Additionally, the internal submerged MBR equipped with quorum quenching microbial vessel showed much lower biofouling than conventional MBR. The quorum quenching effect of the microbial vessel was found to be more pronounced when positioned nearer to the filtration membrane and also depends on recirculation rate of mixed liquor between the bioreactor and membrane tank [2]. It is also observed that the microbial vessel has mentioned its quorum quenching activity steadily over 100 days of MBR operation due to the continuous regeneration of quenching bacteria inside the vessel. This indicates its future potential in designing long-term cost effective antibiofouling strategies in real MBRs treating wastewaters. Recently, a microbial vessel encapsulated with indigenous sludge isolate* Pseudomonas* sp. 1A1 has been found effective in the inhibition of AHL-mediated membrane biofouling in a lab-scale MBR [113]. However, various factors such as vessel material, pore structure, inner volume of vessel, and amount of quorum quenching bacteria have been found to affect the microbial vessel performance and should be taken into account while designing further microbial vessel containing antibiofouling strategies. The microbial vessels have some limitations which need to be resolved before elaborating further for batch scale MBRs, which include the following: (i) as the quorum quenching microbial vessel has been submerged in a fixed place in the MBR, it could degrade only soluble AHLs that were able to diffuse into the vessel, and (ii) the mass transfer of AHLs from the mixed liquor to the inside of the microbial vessel is also limited [148].

To overcome the limitations of quorum quenching microbial vessel, Kim et al. [148] demonstrated cell entrapping beads (CEBs) as an alternative method of bacterial quorum quenching. The CEBs prepared by free-moving beads of alginate entrapped with* Rhodococcus* sp. BH4 have shown the mitigation of membrane biofouling as attributed by both physical (friction) and biological (quorum quenching) effects. The quorum quenching activity of CEBs has also inhibited generation of EPS in biofilm cells and thus formed loosely bound biofilms. This approach of bacterial quorum quenching with CFBs has shown its potential over microbial vessels and found more economically feasible than pure enzymatic quorum quenching. This new process of biofouling control with CFBs could open new horizons in the field of wastewater treatment technology. However, this approach needs further investigation using consortium of quorum quenching bacteria, as real MBR contains diversity of microorganisms which may vary AHLs regulating biofouling phenotype.

## 5. Future Perspectives

The existence of AHL-mediated quorum sensing system in Gram-negative bacteria and its potential role in the formation of biofilms has suggested the application of quorum quenching as an alternative approach for combating membrane biofouling. Recently, some bacterial species having the ability to produce AHL-degrading or modifying enzymes have been identified and successfully attempted in MBRs to reduce biofouling. These evidences strongly indicate that quorum quenching bacteria could be used to develop a potent tool for the control of membrane biofouling. However, the direct application of quorum quenching bacteria has not yet been tried in real MBRs treating municipal or industrial wastewaters. Since wastewaters are composed of diverse groups of biofilm forming bacteria, there is need to design a consortium quorum quenching bacterial system which can destruct a wide range of AHLs and will help to prevent multispecies biofouling in MBR. Additionally, this economically feasible approach needs to be explored further in real MBRs under natural conditions.

## 6. Conclusions

As most of the wastewater bacteria responsible for biofilm formations employ AHL-mediated quorum sensing mechanism to regulate their behaviours, the application of quorum quenching strategy suggests an alternative nontoxic approach for control of biofouling in MBR. The AHLs-mediated quorum quenching mechanisms exist in several Proteobacteria and could be explored further as a new version of antagonism for combating biofilms. Recently, the use of microbial vessel and bead entrapped quorum quenching bacteria has been found as an effective tool in controlling AHL-mediated biofouling in MBRs. These observations will help researchers to design the futuristic AHL-mediated biofouling control strategies in real MBRs treating industrial and municipal wastewaters. Since quorum quenching bacteria showed direct involvement in interfering with quorum sensing behaviours, their further therapeutic and bioindustrial applications should be evaluated in the near future.

## Figures and Tables

**Table 1:** Structures of common *N*-acyl homoserine lactones produced by different Gram-negative bacteria and the phenotype controlled.

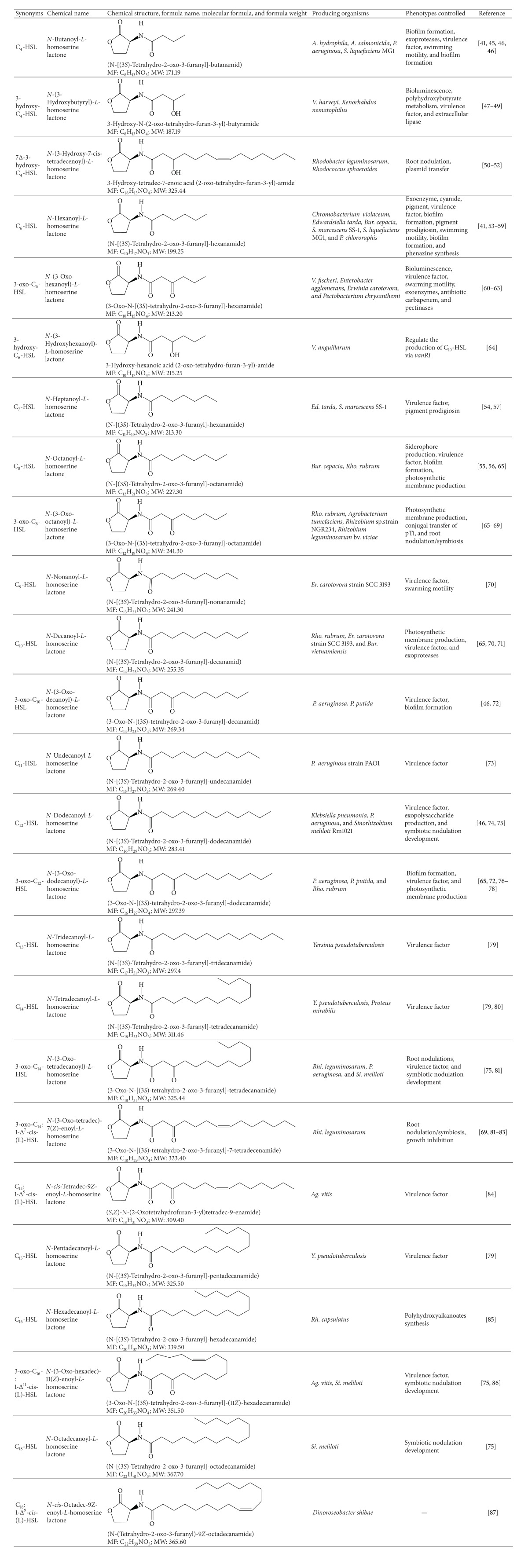

**Table 2:** AHLs producing bacteria present in wastewater treatment systems and quorum sensing phenotypes regulated.

Bacterial strains	AHLs produced	Phenotypes regulated	Reference
*A. hydrophila* subsp. *hydrophila* strain NA1, *A. hydrophila* subsp. *dhakensis *strain LBA2, *A. media* strain NA2, *En. ludwigii* strain SWA1, *K. variicola *strain SWA2, and *S. marcescens* strain SWA6	Short- to medium-chain	Biofilm formation	[88]

*Enterobacter* sp. strain LBA3, *En. cancerogenus* strain LBA4, *Raoultella ornithinolytica* strain TSA7, *P. japonica *strain TSA3, and *Citrobacter freundii* strain R2A5	Long-chain	Biofilm formation	[88]

*A. hydrophila*, *A. media*, *A. punctate*,* A. sobria*,* A. veronii*, *A. jandaei*,* P. oryzihabitans, Ci. farmer*,* Ci. murliniae*, and* En. ludwigii *	Short- to medium-chain	n.d.	[89]

*A. punctata* GC3, *Aeromonas* sp. GC5, *A. hydrophila *GC10, *A. allosaccharophila *GC15,* A. media *GC16,* Citrobacter* sp. GC20, *Acinetobacter johnsonii *GC23,* Klebsiella* sp. GC30,* Shigella* sp. GC37, *Microbacterium paraoxydans *GC42*, Chitinimonas taiwanensis *GC43*, Pantoea agglomerans* GC47*, Ra. terrigena *GC49,and* Microbacterium* sp. GC50	Short- to medium-chain	n.d.	[90]

*A. punctata *GC4,* Aeromonas* sp. GC8, *Aeromonadaceae* sp. GC14, *Citrobacter* sp. GC19, *Neisseria* sp. GC34, *Pseudomonas* sp. GC35, and *Malikia spinosa* GC45	Long-chain	n.d.	[90]

*Ac. junii *	Medium-chain	Biofilm formation	[91, 92]

*A. hydrophila *	Short-chain	n.d.	[93]

*P. putida *	Medium-chain	n.d.	[93]

*Ed. tarda *	Short- and medium-chain	Virulence factor	[54]

n.d.: not determined; short-chain: C_4_-HSL and C_6_-HSL; medium-chain: C_6_-HSL, 3-oxo-C_8_-HSL, and C_8_-HSL; long-chain: C_8_-HSL, 3-oxo-C_8_-HSL, C_10_-HSL, C_12_-HSL, 3-oxo-C_12_-HSL, and C_14_-HSL.

**Table 3:** The biosensors strains developed to detect AHLs produced by Gram-negative bacteria.

Biosensor strain/plasmid	Responded AHLs	Reporter system	Reference
*Ag. tumefaciens* NT1 (pDCI41E33 containing traG::lacZ fusion)	C_6_-HSL, C_8_-HSL, C_10_-HSL, C_12_-HSL, C_14_-HSL, and AHLs with 3-oxo-, 3-hydroxy-, and 3-unsubstituted side chains	*β*-Galactosidase activity	[94]

*Ag. tumefaciens* NT1 (pZLR4 containing Tral/R)	C_6_-HSL, C_8_-HSL, C_10_-HSL, C_12_-HSL, C_14_-HSL, 3-hydroxy-C_6_-HSL, 3-hydroxy-C_8_-HSL, 3-hydroxy-C_10_-HSL, and all AHLs with 3-oxo-side chains	*β*-Galactosidase activity	[95]

*Ag. tumefaciens* A136 (traI-lacZ fusion (pCF218) (pCF372))	C_6_-HSL, C_8_-HSL, 3-oxo-C_8_-HSL, C_10_-HSL, C_12_-HSL, 3-oxo-C_12_-HSL, and C_14_-HSL	*β*-Galactosidase activity	[37]

*C. violaceum* CV026 (CviVR receptor)	C_4_-HSL, C_6_-HSL, and C_8_-HSL	Violacein pigmentation	[53, 96]

*C. violaceum* VIR24 (CviI receptor)	3-Oxo-C_6_-HSL, C_6_-HSL, C_7_-HSL, 3-oxo-C_8_-HSL, C_8_-HSL, C_10_-HSL, C_12_-HSL, and C_14_-HSL	Violacein pigmentation	[97]

*E. coli* (luxCDABE cassette activated by Ahyl/R of *A. hydrophila*)	C_4_-HSL	Bioluminescence	[45]

*E. coli *(pSB401 containing LuxI/R of *V. fischeri*)	C_6_-HSL 3-Oxo-C_8_-HSL C_8_-HSL	luxCDABE	[98]

*E. coli *(pHV2001 containing luxI/R)	C_6_-AHL C_8_-3-oxo-HSL C_8_-HSL	luxCDABE	[99]

*E. coli *(pSB1075 containing LusI/R of *P. aeruginosa*)	3-Oxo-C_12_-HSL, C_12_-HSL	luxCDABE	[98]

*E. coli *(pHV2001-containing LuxI/R of *V. fischeri*)	C_6_-HSL, 3-oxo-C_6_-HSL, 3-oxo-C_8_-HSL, and C_8_-HSL	luxCDABE	[100]

*E. coli *(pKDT17 containing LusI/R of *P. aeruginosa*)	3-Oxo-C_10_-HSL, C_10_-HSL, 3-oxo-C_12_-HSL, and C_12_-HSL	*β*-Galactosidase activity	[100]

*P. putida* 117 (pAS-C8-CepR receptor)	C_8_-HSL	Bioluminescence	[101]

*P. aeruginosa *(M71LZ containing Lasl/R)	3-Oxo-C_10_-HSL, 3-oxo-C_12_-HSL	*β*-Galactosidase activity	[102]

*P. aeruginosa *(pSB406 containing RhlI/R)	C_4_-HSL, C_6_-HSL, C_8_-HSL, C_10_-HSL, C_12_-HSL, and C_14_-HSL with 3-oxo-side chains	luxCDABE	[98]

*P. fluorescens *(pSF105 + pSF107 containing Phzl/R)	3-Hydroxy-C_6_-HSL, 3-hydroxy-C_8_-HSL	*β*-Galactosidase activity	[103]

*Si. meliloti* Rm41 (sinI::lacZ pJNSinR)	C_14_-HSL, 3-oxo-C_14_-HSL, C_16_-HSL, and 1-3-oxo-C_16_-HSL	*β*-Galactosidase activity	[104]

*V. fischeri* (pJBA88 and pJBA89 encoding luxR and Pluxl fusion of gfpmut3∗)	C_6_~C_14_-3-oxo-HSL C_6_~C_12_-HSL	gfp	[105]

**Table 4:** List of quorum quenching bacteria reported to degrade or modify AHLs.

Quenching bacteria	Gene involved	AHLs degraded	Phenotypes regulated	Reference
AHL-acylase mediated QQ				
*Anabaena *sp. PCC7120	*aiiC *	C_4_-HSL, 3-oxo-C_4_-HSL, 3-hydoxo-C_4_-HSL, 3-oxo-C_6_-HSL, 3-hydoxo-C_6_-HSL, C_6_-HSL, 3-oxo-C_8_-HSL, 3-hydoxo-C_8_-HSL, C_8_-HSL, 3-oxo-C_10_-HSL, 3-hydoxo-C_10_-HSL, C_10_-HSL, 3-oxo-C_12_-HSL, 3-hydoxo-C_12_-HSL, C_12_-HSL, 3-oxo-C_14_-HSL, 3-hydoxo-C_14_-HSL, and C_14_-HSL	n.d.	[106]

*Acinetobacter* sp. strain Ooi24	Unknown	C_10_-HSL	n.d.	[89]

*B. pumilus *S8-07	Unknown	3-Oxo-C_12_-HSL	Inhibit biofilm formation in *P. aeruginosa *PA01	[107]

*Comamonas *strain D1	Unknown	C_4_-HSL, 3-oxo-C_6_-HSL, C_6_-HSL, 3-oxo-C_8_-HSL, C_8_-HSL, 3-oxo-C_10_-HSL, C_10_-HSL, 3-oxo-C_12_-HSL, C_12_-HSL, 3-oxo-C_14_-HSL, C_14_-HSL, and C_16_-HSL	Decreases virulence and antibiotic production in *Pec. carotovorum* strain Pcc797	[108]

*P. aeruginosa *	*quiP *	C_6_-HSL, C_8_-HSL, C_10_-HSL, and C_12_-HSL	Inhibits biofilm formation in *Aeromonas*sp.	[109]

*P. aeruginosa *PA01	*PA2385 *	3-Oxo-C_12_-HSL	Reduce virulence factor elastase and pyocyanin in *P. aeruginosa *PA01	[73]

*P. syringae *strain B728a	*hacA *	C_8_-HSL, C_10_-HSL, and C_12_-HSL	Influence biofilm formation	[110]

*P. syringae *strain B728a	*hacB *	3-Oxo-C_6_-HSL, C_6_-HSL, C_8_-HSL, C_10_-HSL, 3-oxo-C_12_-HSL, and C_12_-HSL	Influence biofilm formation	[110]

*P. aeruginosa *PAO1	*PA2385 *	C_11_-HSL, 3-oxo-C_12_-HSL, C_12_-HSL, 3-oxo-C_14_-HSL, and C_14_-HSL	Decreases elastolytic activity and pyocyanin production	[73]

*Pseudomonas* sp. strain PAI-A	*pvdQ *	C_10_-HSL, 3-oxo-C_12_-HSL, C_12_-HSL, and C_14_-HSL	Inhibit virulence factor	[111]

*P. aeruginosa *PAO1	*quiP *	C_8_-HSL, C_10_-HSL, 3-oxo-C_12_-HSL, and C_12_-HSL	Inhibit virulence factor	[112]

*Pseudomonas* sp. 1A1	Unknown	C_6_-HSL, C_8_-HSL, 3-oxo-C_8_-HSL, 3-oxo-C_10_-HSL, C_10_-HSL, 3-oxo-C_12_-HSL, and C_12_-HSL	Inhibit biofilm formation in MBR	[113]

*Rho. erythropolis* strain W2	Unknown	C_4_-HSL, 3-oxo-C_6_-HSL, C_6_-HSL, C_7_-HSL, 3-oxo-C_8_-HSL, C_8_-HSL, and C_10_-HSL	Reduces pathogenicity of *Pec. carotovorum* subsp.* carotovorum *in plants	[114, 115]

*Ralstonia* sp. XJ12B	*aiiD *	3-Oxo-C_8_-HSL, 3-oxo-C_10_-HSL, and 3-oxo-C_12_-HSL	Decreases swarming ability and production of elastase and pyocyanin in *P. aeruginosa* PA01	[20]

*Ralstonia solanacearum *GMI1000	*aac *	C_7_-HSL, C_8_-HSL, 3-oxo-C_8_-HSL, and C_10_-HSL	Inhibits violacein and chitinase activity in* C. violaceum *CV026	[116]

*Streptomyces* sp. strain M664	*ahlM *	C_8_-HSL, C_10_-HSL, and 3-oxo-C_12_-HSL	Decreases virulence factor, elastase, protease, and LasA in *P. aeruginosa *	[117]

*Shewanella* sp. strain MIB015	*aac *	C_8_-HSL, C_10_-HSL, and C_12_-HSL	Reduces biofilm formation in *V. anguillarum *	[118]

*Variovorax paradoxus* strain VAI-C	Unknown	C_4_-HSL, 3-oxo-C_6_-HSL, C_6_-HSL, C_8_-HSL, C_10_-HSL, and C_12_-HSL	n.d.	[119]

AHL-lactonase mediated QQ				
*Ag. tumefaciens* c58	*attM *	3-Oxo-C_8_-HSL	Inhibit Ti plasmid conjugal transfer	[120]

*Ag. tumefaciens *	*aiiB *	C_4_-HSL, 3-oxo-C_6_-HSL, C_6_-HSL, 3-oxo-C_8_-HSL, C_8_-HSL, and C_10_-HSL	n.d.	[121]

*Ag. tumefaciens* C58	*aiiB *	3-Oxo-C_6_-HSL, C_6_-HSL, C_8_-HSL, C_7_-HSL, 3-oxo-C_8_-HSL, and C_8_-HSL	Reduces virulence of *Erwinia *strain 6276	[122]

*Ag. tumefaciens* K84	*aiiS *	3-Oxo-C_6_-HSL, C_6_-HSL, 3-oxo-C_8_-HSL, C_8_-HSL, 3-oxo-C_10_-HSL, C_10_-HSL, 3-oxo-C_12_-HSL, C_12_-HSL, 3-oxo-C_14_-HSL, and C_14_-HSL	n.d.	[123, 124]

*Acinetobacter* sp. strain C1010	Unknown	C_6_-HSL, C_8_-HSL	Inhibit production of phenazines in *P. chlororaphis* O6 and virulence in *Er. carotovora *	[125]

*Acinetobacter* sp. GG2	Unknown	3-Hydroxy-C_4_-HSL, C_5_-HSL, 3-hydroxy-C_6_-HSL, C_6_-HSL, C_7_-HSL, 3-oxo-C_8_-HSL, 3-hydroxy-C_8_-HSL, C_8_-HSL, C_9_-HSL, and 3-oxo-C_10_-HSL, 3-hydroxy-C_10_-HSL, C_10_-HSL, C_11_-HSL, 3-oxo-C_12_-HSL, 3-hydroxy-C_12_-HSL, C_12_-HSL, 3-oxo-C_14_-HSL, 3-hydroxy-C_14_-HSL, C_14_-HSL, Δ^9^-3-hydroxy-C_14_-HSL, Δ^10^-3-hydroxy-C_14_-HSL, Δ^11^-3-hydroxy-C_14_-HSL, and Δ^13^-3-hydroxy-C_14_-HSL	Attenuates virulence of *P. aeruginosa* and *Er. carotovora *	[109]

Acidobacteria sp.	*qIcA *	3-Oxo-C_6_-HSL, C_6_-HSL, C_7_-HSL, 3-oxo-C_8_-HSL, C_8_-HSL, 3-oxo-C_10_-HSL, and C_10_-HSL	Decreases virulence of *Pec. carotovorum *strain 6276	[126]

*Arthrobacter* sp. IBN110	*ahlD *	C_4_-HSL, 3-oxo-C_6_-HSL, C_6_-HSL, C_8_-HSL, 3-oxo-C_10_-HSL, and C_10_-HSL	Decreases virulence of *Er. carotovora *N98	[127]

*Bacillus* sp. 240B1	*aiiA *	C_8_-HSL	Decreases extracellular pectolytic enzyme activities and inhibits virulence in *Er. carotovora *	[128, 129]

*B. cereus *	*aiiA *	C_6_-HSL, C_8_-HSL, and C_10_-HSL	Decreases virulence factor	[130]

*B. mycoides *	*aiiA *	C_6_-HSL, C_8_-HSL, and C_10_-HSL	Decreases virulence factor	[130]

*Bacillus* strain COT1	*aiiA *	3-Oxo-C_6_-HSL	Decreases virulence factor	[130]

*B. anthracis *	*aiiA *	C_6_-HSL, C_8_-HSL, and C_10_-HSL	Decreases swarming in *Bur. thailandensis *	[131]

*B. pumilus *SW9	Unknown	n.d.	Inhibit biofouling on microfiltration membranes by *Brevundimonas* sp. SW1, *Acidovorax* sp. DB3, *Acinetobacter* sp. GS1, and *Staphylococcus aureus* SA1	[132, 133]

*B. thuringiensis* subspecies *morrisoni *	*aiiA *	3-Oxo-C_6_-HSL	Attenuates the pathogenicity of *Er. carotovora *	[134]

*B. thuringiensis *	*aiiA *	3-Oxo-C_6_-HSL	Decreases virulence of *Er. carotovora *	[135]

*Bacillus* sp. A24	*aiiA *	C_4_-HSL, C_6_-HSL	Decreases production of elastase, rhamnolipids, and pyocyanin and inhibits swarming in *P. aeruginosa *PA01	[136]

*En. asburiae *VT65	*aiiA *	C_4_-HSL, C_6_-HSL	n.d.	[137]

*Geobacillus kaustophilus* strain HTA426	*GKL *	C_4_-HSL, 3-oxo-C_6_-HSL, C_6_-HSL, 3-oxo-C_8_-HSL, C_8_-HSL, C_10_-HSL, and 3-oxo-C_12_-HSL	Thermostable antivirulence therapeutic agent	[138]

*K. pneumonia *	*ahlK *	C_6_-HSL, 3-oxo-C_6_-HSL	Decreases virulence of *Er. carotovora *N98	[127]

*M. testaceum *StLB018	Unknown	C_6_-HSL, 3-oxo-C_6_-HSL, C_10_-HSL, and 3-oxo-C_10_-HSL	Interrupts pathogenicity of *Pec. carotovorum* subsp.* carotovorum *	[139]

*Mycobacterium avium* subsp. *paratuberculosis *K-10	*MCP *	C_7_-HSL, C_8_-HSL, 3-oxo-C_8_-HSL, C_10_-HSL, and C_12_-HSL	n.d.	[140]

*My. tuberculosis *	*AhlA, PPH *	C_4_-HSL, 3-oxo-C_8_-HSL, and C_10_-HSL	n.d.	[141]

*M. testaceum *StLB037	*aiiM *	3-Oxo-C_6_-HSL, C_6_-HSL, 3-oxo-C_8_-HSL, C_8_-HSL, 3-oxo-C_10_-HSL, and C_10_-HSL	Reduces pectinase activity and virulence in *Pec. carotovorum* subsp. *carotovorum *	[142]

*Ochrobactrum* sp. T63	*aidH *	C_4_-HSL, C_6_-HSL, 3-oxo-C_6_-HSL, 3-oxo-C_8_-HSL, and C_10_-HSL	Reduce biofilm formation by *P. fluorescens* 2P24 and the pathogenicity of *Pec. carotovorum *	[143]

*Pichia pastoris *	*aiiA*_ B546_	3-Oxo-C_6_-HSL, C_6_-HSL, 3-oxo-C_8_-HSL, C_8_-HSL, C_10_-HSL, and C_12_-HSL	Attenuates the *A. hydrophila* infection in aquaculture	[144]

*Pseudoalteromonas byunsanensis* strain 1A01261	*qsdH *	C_4_-HSL, 3-oxo-C_6_-HSL, C_6_-HSL, 3-oxo-C_8_-HSL, C_8_-HSL, C_10_-HSL, C_12_-HSL, and C_14_-HSL	Attenuates the plant pathogenicity of *Er. carotovora *	[145]

*Rho. erythropolis *W2	*qsdA *	3-Oxo-C_6_-HSL, C_6_-HSL, 3-oxo-C_8_-HSL, C_8_-HSL, 3-oxo-C_10_-HSL, C_10_-HSL, 3-oxo-C_12_-HSL, C_12_-HSL, 3-oxo-C_14_-HSL, and C_14_-HSL	Decreases virulence of *Pec. carotovorum *strain PCC797	[146]

*Rhodococcus *strain LS31	Unknown	C_6_-HSL, 3-oxo-C_6_-HSL, C_10_-HSL, and 3-oxo-C_10_-HSL	Reduces pectate lyase activity in *Er. carotovora *	[147]

*Rhodococcus *strain PI33	Unknown	C_6_-HSL, C_10_-HSL	Reduces pectate lyase activity in *Er. carotovora *	[147]

*Rhodococcus* sp. BH4	*qsdA *	3-Oxo-C_6_-HSL, C_6_-HSL, 3-oxo-C_8_-HSL, C_8_-HSL, 3-oxo-C_10_-HSL, C_10_-HSL, 3-oxo-C_12_-HSL, and C_12_-HSL	Inhibit biofilm formation in MBR	[148, 149]

*Rhodococcus* sp. A167	Unknown	C_6_-HSL, 3-oxo-C_8_-HSL, and C_8_-HSL	Attenuates maceration ability of *Pec. carotovorum* subsp. *carotovorum *	[150]

*Solibacillus silvestris *StLB046	*ahlS *	C_10_-HSL	Attenuates maceration of plant pathogen *Pec. carotovorum* subsp. *carotovorum *	[151]

*Thalassomonas* sp. PP2-459	Unknown	C_4_-HSL, C_6_-HSL, C_8_-HSL, 3-oxo-C_10_-HSL, C_10_-HSL, and C_12_-HSL	Decreases pathogenicity of *V. anguillarum* ATCC 19264	[152]

Oxidoreductase mediated QQ				
*B. megaterium* CYP102A1	*P450BM3 *	Oxidizes; C_12_-HSL, 3-oxo-C_12_-HSL, C_14_-HSL, 3-oxo-C_14_-HSL, C_16_-HSL, C_18_-HSL, and C_20_-HSL.	n.d.	[153]

*Rho. erythropolis *W2	Unknown	Oxidizes; 3-oxo-C_10_, 3-oxo-C_12_-HSL	n.d.	[115]

n.d.: not determined.

## Abbreviations

*Ac.*: * Acinetobacter*
*A.*: * Aeromonas*
*Ag.*: * Agrobacterium*
*B.*: * Bacillus*
*Bur.*: * Burkholderia*
*C.*: * Chromobacterium*
*Ci.*: * Citrobacter*
*Ed.*: * Edwardsiella*
*E.*: * Escherichia*
*En.*: * Enterobacter*
*Er.*: * Erwinia*
*K.*: * Klebsiella*
*M.*: * Microbacterium*
*My.*: * Mycobacterium*
*Pec.*: * Pectobacterium*
*P.*: * Pseudomonas*
*R.*: * Ralstonia*
*Ra.*: * Raoultella*
*Rhi.*: * Rhizobium*
*Rh.*: * Rhodobacter*
*Rho.*: * Rhodococcus*
*S.*: * Serratia*
*Si.*: * Sinorhizobium*
*V.*: * Vibrio*
*Y.*: * Yersinia*
AHL: * N*-Acyl homoserine lactone(s)
HSL: Homoserine lactone.
